# Prognostic Evaluation of Metastasis-Related Lymphocyte/Monocyte Ratio in Stage Ⅰ-Ⅲ Breast Cancer Receiving Chemotherapy

**DOI:** 10.3389/fonc.2021.782383

**Published:** 2022-03-24

**Authors:** Zihan Zhang, Qian Lin, Yi Chen, Chenlin Su, Wuye Lin, Daoyu Wei, Litu Zhang, Haizhou Liu

**Affiliations:** ^1^Department of Research, Guangxi Medical University Cancer Hospital, Nanning, China; ^2^Development Planning Office, Guangxi Medical University , Nanning, China; ^3^Department of Research, Guangxi Cancer Molecular Medicine Engineering Research Center, Nanning, China

**Keywords:** breast cancer, predictive factor, lymphocyte/monocyte ratio, inflammatory biomarkers, prognostic

## Abstract

**Purpose:**

This study aims to clarify the prognostic significance of metastasis-related indicators in peripheral blood in stage I-III breast cancer (BC).

**Methods:**

The clinicopathological data of 938 breast cancer patients and 509 benign breast disease patients were retrospectively analyzed, and fasting blood samples were collected before treatment. Univariate and multivariate regression analyses were used to evaluate factors related to metastasis risk and prognosis. The Kaplan-Meier method was used to generate survival curves, and the log-rank test was used to measure differences in survival between groups.

**Results:**

Use the cut-off value (3.433) of LMR, the logistic regression analysis revealed that high carbohydrate antigen 153 (CA153), carbohydrate antigen 125 (CA125), carcinoembryonic antigen (CEA), killer T cell level, and low lymphocyte to monocyte ratio (LMR) level were significantly associated with BC distant metastasis. In contrast, LMR>=3.433 (HR: 0.409, 95%CI: 0.193–0.867, P = 0.020), Th/Tc ratio >=1.946 (HR: 0.378, 95% CI: 0.158–0.904, P =0.029) is regarded as a protective factor in the multivariate cox analyses. LMR is an independent prognostic factor for DFS in HER2-negative BC patients.

**Conclusion:**

Peripheral blood parameters play an important role in predicting distant metastasis and prognosis of BC patients. As a potential marker, LMR can predict the metastasis and prognosis of patients with stage I-III BC.

## Introduction

Breast cancer (BC) is the most common cancer in women and the leading cause of cancer death among them. In both sexes combined, female BC (11.6% of all cases) is the second most widely diagnosed cancer in 20 regions of the world (1). Curative surgical treatment of local BC patients and pathological sampling of lymph nodes are the first steps in treatment in the traditional sense (2). However, in the current treatment, neoadjuvant chemotherapy before surgery has been considered the preferred strategy for operable or non-operable BC (3). Recurrence or metastasis may occur in these treated BC patients, but we lack effective and reliable predictive biomarkers to guide risk stratification before treatment (4, 5). However, these studies’ results are still inconsistent in the efficacy of risk estimation among various tumors, and it is difficult to find accurate estimates of BC diagnosis and metastasis.

Immune function is an important prognostic factor of BC. It is involved in tumorigenesis, progression, and metastasis (6). Platelet to lymphocyte ratio (PLR), lymphocyte to monocyte ratio (LMR), neutrophil to lymphocyte ratio (NLR) in tumors, including BC, shows its diagnostic and prognostic value (7–9). We observed changes in the ratio of neutrophils, lymphocytes, and monocytes in peripheral blood during the tumor immunity response (10, 11). In these studies, the percentage of NLR is associated with a poor tumor prognosis (12). Changes in the ratio of LMR and PLR also reflect that the balance between the adaptive immune system and the innate immune system is broken, and the body lacks anti-tumor activity (13, 14)

Currently, research shows that the lymphocyte to monocyte ratio (LMR) can be used as a biomarker for BC detection and monitoring (15, 16). More and more reports indicate that LMR is used in neoadjuvant chemotherapy for various tumors and is a powerful biomarker to verify the efficacy (17–19). All patients need to check the peripheral blood before treatment, and the indicators are cheap and easy to obtain.

Although some studies use the ROC curve based on DFS/OS to determine the LMR cut-off value (20), the DFS/OS outcome is sometimes too subjective and requires a lot of follow-up work, so the cut-off value of different studies is quite different. As we all know, patients with stage IV metastatic breast cancer generally have poor outcomes. Therefore, we used the blood parameters and cut-off ratio points determined by the ROC curve related to distant metastasis to retrospectively analyze the relationship between the survival status of BC patients after treatment and the LMR before treatment. We aim to explain the diagnosis value of these biomarkers as pre-treatment variables and test whether these biomarkers can also be used as post-treatment surveillance parameters.

## Method

### Two Distinct Cohorts Composed the Comprehensive Study

Our study retrospectively collected data on patients who were treated at Guangxi Medical University from May 2018 to May 2020, the inclusion criteria as follows: 1) woman with BC or breast benign disease, which was confirmed by histology; 2) BC has to be primary; 3) the BC patients’ clinical features, hematological indicators, and inflammatory biomarkers are complete, and 4) the breast benign disease patients’ clinical features and hematological indicators are complete. The exclusion criteria were as follow: 1) patients with other malignant tumors; 2) patients who have acute or chronic hematologic disease, severe systemic infection, or autoimmune diseases; 3) the patients’ clinical features and indicators required by the study are incomplete.

A total of 938 patients who received standard treatment and did not receive anticancer therapy before their surgery enrolled in the study. Patients were followed up for at least 0.5 months. The control cohort consisted of 509 female patients with benign breast diseases treated in our hospital. The laboratory medicine department carries out inflammatory biomarkers. This research was conducted in the Guangxi Medical University Cancer Hospital; this study followed the 2008 Declaration of Helsinki’s ethical guidelines and our hospital code of ethics (LW2021086).

### Data Collection

Participants’ data is divided into three parts: clinical-pathological features, blood system indicators, and immunological indicators. The clinical pathological features included age, estrogen receptor (ER), progesterone receptor (PR), HER2, Ki-67, CK5-6, epidermal growth factor receptor (EGFR), TNM stage, location, and transfer area. The blood system indicators contained carcinoembryonic antigen (CEA), carbohydrate antigen 125 (CA125), carbohydrate antigen 153 (CA153) (21, 22), neutrophile, monocyte, platelet. Furthermore, the immunological indicators involved T cell, helper T cell (Th), killer T cell (Tc), natural killer (NK) cell, and B cell (23). All blood samples were collected before treatment. The prognosis was obtained through the follow-up department of this hospital and telephone.

### Serum and Plasma Tumor-Related Markers

Before treatment, we collected 3ml of peripheral venous blood from all patients. Serum CEA, CA125, and CA153 were measured using an automatic chemiluminescence immunoassay system (SIEMENS ADVIA centaur; Siemens, Germany). NLR was the absolute neutrophil count/the total lymphocyte, LMR was the total lymphocyte/the absolute monocyte, and PLR was the total platelet count/the total lymphocyte count. These parameters were analyzed from the peripheral blood cell count (DxH 800 hematology analyzer, Beckman Coulter). See **Supplementary** for the experimental methods of cellular immunology related indicators.

### Statistical Analysis

The characteristics of BC patients and benign breast diseases were summarized and described. Frequency distribution represents categorical variables, whereas continuous variables are reported through the median and interquartile range. LMR, NLR, PLR, Th/Tc ratios were calculated from raw hematology indicators. Markers with disease and clinicopathological features were explored by Wilcoxon rank-sum test or Kruskal–Wallis test. The receiver operating characteristic (ROC) curve is built for selecting the optimal threshold and diagnostic accuracy of these continuous indicators. The least absolute shrinkage and selection operator (LASSO) (24) regression model and the multivariable logistic and Cox regression analysis were used to establish a convincing prediction model in BC patients. Disease-Free Survival (DFS) was the time between treatment start and disease recurrence or patient death due to disease progression. Survival difference analysis between groups used by log-rank test and display with the Kaplan-Meier plot. R-4.04 software was used for statistical analysis.

## Result

### Clinicopathological Features and Inflammatory Biomarkers

**Figure 1:**
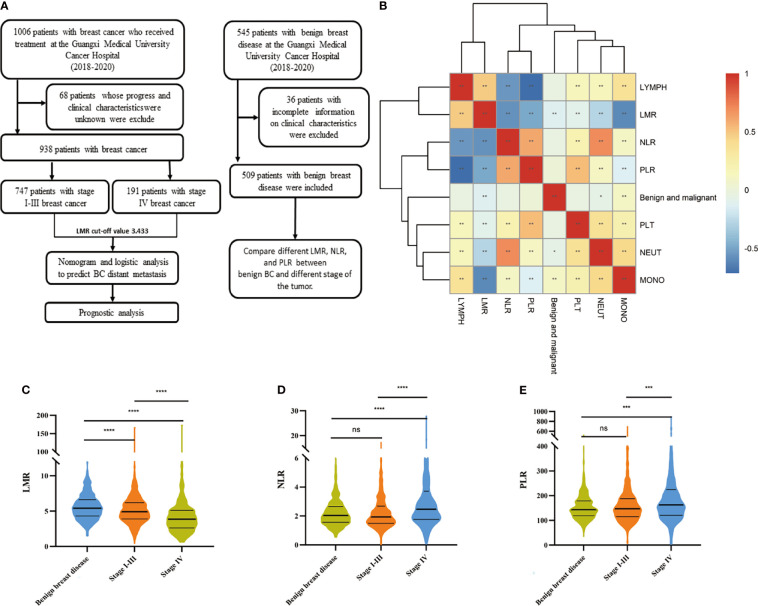
Working mode and Distribution of LMR and other indicators in benign breast diseases and tumors. **(A)** The workflow of this study. **(B)** Visualization of the correlation matrix of hematology test indicators and benign and malignant tumors, showing that MONO, NRUT, and LMR are related to tumor malignancy. Boxplot of **(C)** LMR **(D)** NLR and **(E)** PLR in benign BC and different stages of the tumor. Differences between groups estimated by Mann–Whitney U as appropriate. (*P < 0.05, **P < 0.01, ***P < 0.001, ****P < 0.0001, ns P > 0.05).

In this study, 938 BC patients and 509 benign breast disease patients were included after applying the conditions (**Figure 1A**), and all participants were women. The median age of benign breast disease was 37 years, and the BC patients were 49 years. There were 747 patients diagnosed as stage I-III and 191 patients diagnosed as stage IV among BC patients.

PR-positive accounted for 67.70% of all BC patients, and ER-positive accounted for 71.32%. Of all BC patients 48.93% were HER2 positive, 12.90% CK5-6 positive, and 17.80% were EGFR positive. A further 82.94% of patients of Ki-67 were higher than 14%. Based on the above results, BC patients were identified as luminal A, luminal B, ERBB2, and triple-negative BC (10.13%, 66.31%, 12.69%, and 10.87%).

The median values of the NLR, PLR, LMR were 2.04 [1.56,2.86], 4.75 [3.66,6.08], 150.17 [116.71,194.76] in BC patients. the median values of the NLR, PLR, LMR were 2.04 [1.56,2.65], 5.42 [4.32,6.60], 143.65 [118.75,179.08] in benign breast disease patients. The patients’ details are list in **Table 1**.

**Table 1:** Clinicopathological factors and baseline characteristics.

Characteristics	BC	Benign breast disease	*P-*value
Number of patients	938	509	
Age (years)			<0.01
Average (SD)	49.63 (11.07)	37.49 (11.91)	
Median (IQR)	49 (42,56)	37 (29,45)	
<45 years (%)	309 (32.94%)	375 (73.67%)	
≱ 45 to <55 years (%)	346 (36.89%)	92 (18.07%)	
≱ 55 years (%)	283 (30.17%)	42 (8.25%)	
T classification			
T_1_T_2_	671 (71.54%)	/	
T_3_T_4_	267 (28.46%)	/	
N classification			
N_0_	410 (43.71%)	/	
N_1-3_	528 (56.29%)	/	
M classification			
M_0_	747 (79.64%)	/	
M_1_	191 (20.36%)	/	
Clinical stage			
I/II	554 (59.06%)	/	
III/IV	384 (40.94%)	/	
Predictive factors			
Progesterone Receptor + (%)	635 (67.70%)	/	
Estrogen Receptor + (%)	669 (71.32%)	/	
HER2+ (%)	459 (48.93%)	/	
Ki_67≧14% (%)	778 (82.94%)	/	
CK5_6 + (%)	121 (12.90%)	/	
EGRF + (%)	167 (17.80%)	/	
Molecular subtypes			/
Luminal A	95 (10.13%)	/	/
Luminal B(HER2(-))	282 (30.06%)	/	/
Luminal B(HER2(+))	340 (36.25%)	/	/
ERBB2(HER2(+))	119 (12.69%)	/	/
Triple‐negative	102 (10.87%)	/	/
Metastatic and relapse sites			
liver metastasis (%)	16 (1.71%)	/	/
lung metastasis (%)	3 (0.03%)	/	/
distant lymph node (%)	5 (0.05%)	/	/
others (%)	71 (7.57%)	/	/
multiple metastasis (%)	96 (10.23%)	/	/
CEA (median [IQR])	1.84 [1.22,3.07]	/	/
CA125 (median [IQR])	15.30 [10.10,24.98]	/	/
CA153 (median [IQR])	13.80 [8.10,22.80]	/	/
T cell (median [IQR])	67.50 [61.50,73.30]	/	/
Th (median [IQR])	39.40 [33.73,44.50]	/	/
Tc (median [IQR])	20.77 [16.70,25.58]	/	/
Th/Tc ratio(median [IQR])	1.90[1.44,2.50]	/	/
NK (median [IQR])	11.90 [8.40,17.00]	/	/
B cell (median [IQR])	12.33 [9.02,16.00]	/	/
**NEUT(median [IQR])**	**3.62 [2.85,4.64]**	**3.72 [2.92,4.78]**	**0.023**
LYMPH (median [IQR])	1.75 [1.40,2.12]	1.84 [1.53,2.11]	0.118
**MONO (median [IQR])**	**0.38 [0.30,0.46]**	**0.34 [0.28,0.42]**	**<0.01**
PLT (median [IQR])	259.00 [220.00,310.00]	268.00 [232.00,309.00]	0.139
NLR (median [IQR])	2.04 [1.56,2.86]	2.04 [1.56,2.65]	0.239
**LMR (median [IQR])**	**4.75 [3.66,6.08]**	**5.42 [4.32,6.60]**	**<0.01**
PLR (median [IQR])	150.17 [116.71,194.76]	143.65 [118.75,179.08]	0.603

Wilcoxon test. Bold values indicate significant differences.

EGRF, epidermal growth factor receptor; CEA, carcinoembryonic antigen; CA125, carbohydrate antigen 125; CA153, carbohydrate antigen 153; Th, helper T cell; Tc, killer T cell; NK, natural killer; NEUT, neutrophile; LYMPH, lymphocyte; MONO, monocyte; PLT, platelet; NLR, neutrophil-lymphocyte ratio; LMR, lymphocyte-monocyte ratio; PLR, platelet-lymphocyte ratio.

### Compared With Benign Breast Disease and BC Patient Group in Inflammatory Biomarkers

The heat map shows that LMR is associated with benign breast disease and malignant BC (**Figure 1B**). Moreover, in the analyses of tumors benign and malignant, the level of LMR is highest in patients with benign breast disease. It gradually decreases in patients with stage I-III BC and stage IV BC, as shown in **Figure 1C** (P<0.01). The NLR and PLR gradually increased in patients with stage I-III BC and stage IV BC, but the difference between stage I-III BC patients and benign BC was not significant, as shown in **Figures 1D, E**.

### Optimal Threshold of Inflammatory Biomarkers in BC

**Figure 2:**
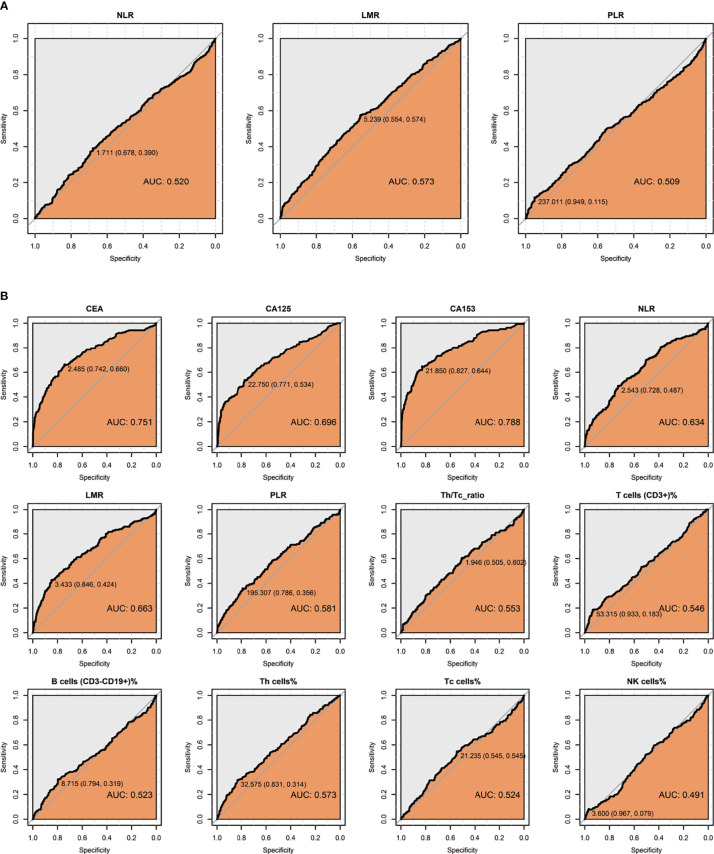
ROC curve of peripheral blood indicators. **(A)** Perform ROC curve analysis on 1483 breast disease patients to select the best hematological index boundary value for distinguishing BC, including NLR, LMR, and PLR. **(B)** Performed on 938 BC patients to select the best cut-off value of hematological parameters for distinguishing patients with distant metastasis of BC, including CA153, CEA, CA125, NLR, LMR, PLR, Th cell%, Th/Tc radio, T cell%, Tc cell%, B cell%, NK cell%.

ROC curves were analyzed the optimal threshold and their diagnostic sensitivities and specificities drawn in **Figure 2** and **Table S1**. For the diagnosis of BC, we found that peripheral blood NLR, LMR, and PLR have a low diagnostic value (AUC<0.6) in BC with the threshold as 1.711, 5.239, and 237.011. When using one marker in BC distant metastasis, the best sensitivity was CEA (0.66), the best specificity was LMR with 0.846, and the highest Youden’s index was CA153, respectively. Using the cut-off values in **Table S1**, we divided BC into high and low groups according to different markers for subsequent analysis.

### Correlation Between Pre-Therapeutic Inflammatory Biomarkers With Clinicopathological Data

**Figure 3:**
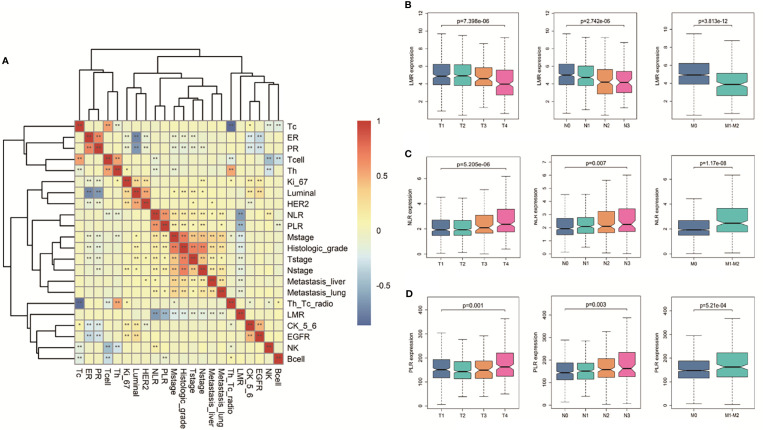
Correlation between clinical indicators and hematological indicators. **(A)** The heat map of the correlation between clinical indicators and hematological indicators in BC. Different colors in the figure correspond to different correlation coefficients, and different significance levels are marked as *P < 0.05, **P < 0.01. **(B)** Boxplots of the significantly reduced LMR in the clinical TNM stage. Boxplots of elevated **(C)** NLR and **(D)** PLR in the clinical TNM setting. Differences between groups were estimated using the Kruskal–Wallis test.

In **Figure 3A**, we initially compared the correlations between different markers and pathological data and traditional immune index, including Th, Tc, Bcell, etc. We found that LMR has no correlation with immune indicators, but it is related to metastasis and stage. At the first diagnosis, we divide BC patients into multiple groups based on demographic and clinical characteristics collected before receiving any treatment to assess differences in the baseline concentration of different markers. As shown in **Table S2**, most patients with advanced BC have high NLR, PLR, and low LMR ratios (p<0.001). The Kruskal–Wallis test of the TNM stage revealed that high PLR, NLR, and low LMR correlated with high T stage, lymph node metastasis, and distant metastasis, shown in **Figures 3B–D**.

### The Predictive Value of Inflammatory Biomarkers for BC Distant Metastasis

**Figure 4:**
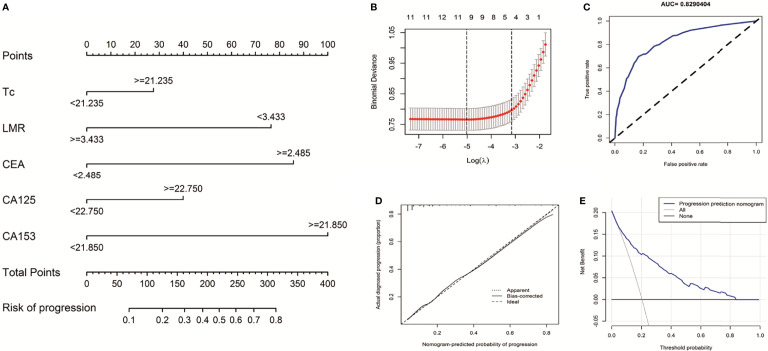
Nomogram for prediction of BC metastasis risk. **(A)** shown is the Nomogram for predicting the risk of stage IV based on Multivariate logistic regression analysis in BC patients. **(B)** Selection results of the LASSO model. The partial likelihood deviance (binomial deviance) curve plot versus logλ. Dotted vertical lines draw at the optimal values by using the minimum criteria. **(C)** Plotted is a ROC curve for the independent validation cohort of the logistic regression model used in the Nomogram. The AUC is denoted, with the closer to 1, the better the model is. **(D)** the calibration plot gives the prediction performance of the proposed Nomogram in the discovery cohort, with the closer to the 45° line, the better the performance. **(E)** Decision curve analysis for the risk nomogram. The X-axis is the risk threshold probability that changes from 0 to 1, and the Y-axis is the calculated net benefit for a given threshold probability.

To explore the diagnostic significance of high LMR before treatment for BC distant metastasis, we used five peripheral blood indicators to construct a nomogram to predict BC metastasis. These indicators are the smallest P values in multiple logistic regression (**Figure 4A**). First, based on 938 BC patients, we used LASSO and 10-fold cross-validation to screen out nine indicators (**Figure S1**), included Tcell, helper T cell, killer T cell, B cell, NLR, LMR, CEA, CA125, and CA153 (**Figure 4B**, lambda.min=0.006644068). To further verify the accuracy of the results, the univariate and multivariate logistic regression analysis among these above features is shown in **Table 2**. The model finally contains CA153 (OR=4.307, P<0.001), LMR (OR=0.375, P<0.001), CEA (OR=3.345, P<0.001), CA125 (OR=1.625, P=0.021), and killer T cell (OR=1.700, P=0.012). The BC metastasis risk nomogram’s calibration curve, which use to predict a BC patient’s risk of progressing to stage IV, shows excellent consistency (**Figure 4C**, AUC=0.8290404). The model was verified by bootstrapping (**Figure 4D**, C-index=0.829). The model decision curve analysis showed that using a nomogram to predict BC metastasis risk would benefit more than the scheme if the threshold probability of patients and doctors are >4% and <84% (**Figure 4E**), respectively.

**Table 2:** Univariate and multivariate logistic regression analysis predicts distant metastasis of BC.

Univariate					Multivariate			
Profiles	OR	95 (%) CI		p	OR	95 (%) CI		p
CA153>=21.850	8.666	6.119	12.374	<0.001	4.307	2.863	6.499	<0.001
CEA>=2.485	5.564	3.971	7.863	<0.001	3.345	2.248	4.996	<0.001
LMR>=3.433	0.247	0.174	0.350	<0.001	0.375	0.227	0.618	<0.001
Tc>=21.235	1.431	1.041	1.972	0.028	1.700	1.130	2.577	0.012
CA125>=22.750	3.860	2.774	5.387	<0.001	1.625	1.073	2.444	0.021
Th/Tc ratio >=1.946	0.649	0.468	0.894	0.009				
Th>=32.575	0.443	0.310	0.638	<0.001				
Tcell>=53.315	0.320	0.201	0.512	<0.001				
PLR>=195.307	2.028	1.434	2.855	<0.001				
NLR>=2.543	2.543	1.835	3.526	<0.001				
NK>=3.6	0.406	0.212	0.804	0.008				
Bcell>=8.715	0.553	0.390	0.790	0.001				

CEA, carcinoembryonic antigen; CA125, carbohydrate antigen 125; CA153, carbohydrate antigen 153; NK, natural killer; NLR, neutrophil to lymphocyte ratio; PLR, platelet to lymphocyte ratio; LMR, lymphocyte to monocyte ratio; Th/Tc ratio, helper T cell to killer T cell ratio; CI, confidence interval; OR, odds ratio.

### Low LMR Indicated Poor Prognosis in Stage Ⅰ-Ⅲ BC

**Figure 5:**
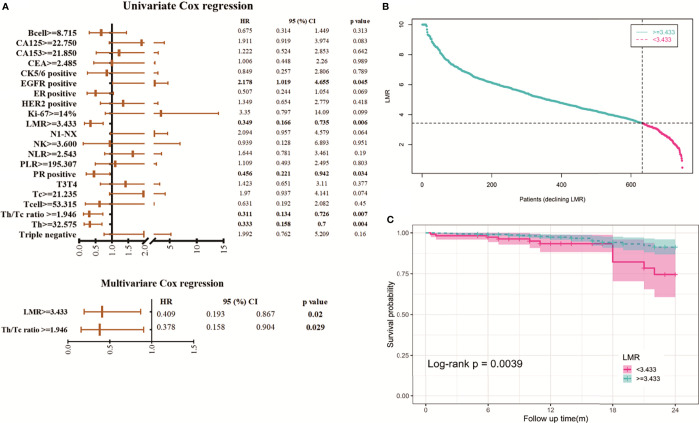
Assessment of the prognostic risk model of LMR and clinical features in stage I-III BC. **(A)** Univariate and multivariate analyses of the clinical characteristics and LMR with the DFS. **(B)** Distribution of LMR risk score for the stage I-III BC. **(C)** Kaplan-Meier survival curves of the DFS of patients in the different LMR groups for stage I-III BC.

To explore the clinical significance of high LMR before treatment for BC, in our research cohort, the median follow-up period of 12 months (range: 0.5-24 months) of 747 stage I-III BC patients, 30 patients (4%) experienced disease recurrence or died (for any reason). **Table S1** determines the cut-off value of our grouping here. Univariate and multivariate Cox proportional hazards models conduct to investigate the relationship between clinical variables and DFS (**Figure 5A**). In the multivariate analysis, high LMR (HR=0.409, p=0.02) and Th/Tc ratio (HR=0.378, p=0.029) were independent prognostic factors of a protective factor in stage I-III BC. The time-dependent ROC curve analysis of LMR shows that the maximum AUC of our LMR in 6-24 months is 0.649 (**Figure S2**). We further analyze the distribution of risk levels, survival status, and survival time patterns of BC patients with different LMR (**Figures 5B**). Kaplan-Meier survival analyses performed on the stage I-III BC showed the DFS of BC patients with lower LMR values was worse than that of patients with higher LMR values (**Figures 5C**). We regret that no clinically valuable markers have been screened in either univariate or multivariate survival analysis in stage IV BC. The results show in **Table S3**.

### Clinical Prognostic Evaluation of LMR in Different Molecular Subtypes of BC

**Figure 6:**
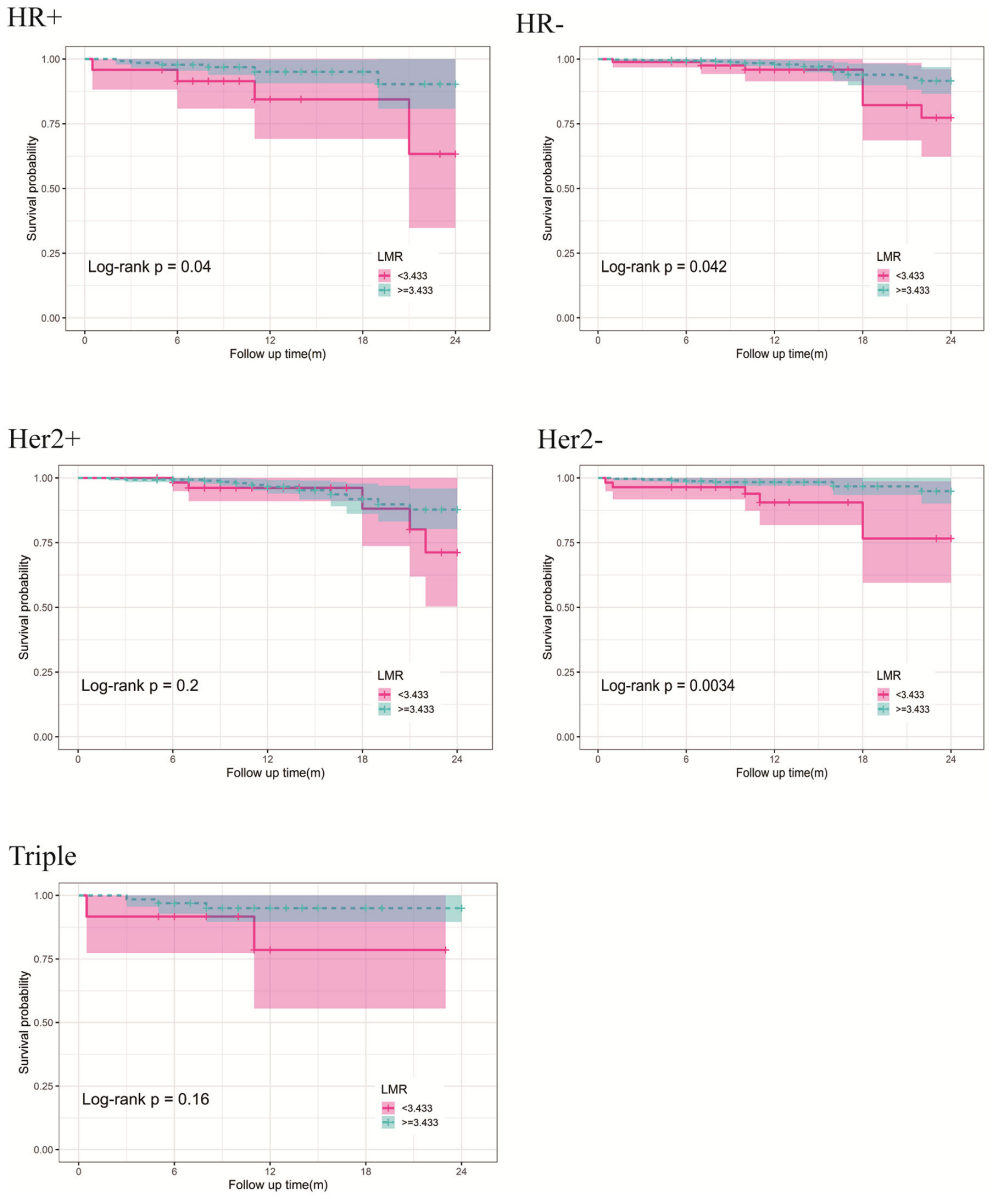
Kaplan-Meier curves of DFS differences stratified by the molecular subtypes between the high- and low-LMR groups in the stage I-III BC.

We retrospectively analyzed the relationship between preoperative blood parameters and clinical outcomes after treatment in BC patients with different molecular subtypes. According to HR (ER, PR) expression, BC patients are divided into HR-positive (either ER, PR are positive for any term) and HR negative groups. Patients are divided into HER-2 positive and HER-2 negative groups according to HER-2 expression. In addition, we also discussed patients with triple-negative BC. Although in univariate analysis, LMR is a prognostic factor for DFS in HR-positive (p = 0.048), HR-negative (0.054), and HER2-negative (p = 0.007) BC patients, it is only an independent prognostic factor for DFS in HER2-negative BC patients (LMR, P = 0.022). We did not find any indicators as independent prognostic factors for DFS in patients with HR negative and triple-negative BC. The results are presented in **Table S4**. Using the cut-off values in **Table S1** divided stage I-III BC. The discrepancies in DFS stratified by the molecular subtypes were analyzed. The DFS of the high-LMR group was still better than the low LMR group, except for HR-positive and triple-negative subtypes (**Figure 6**).

## Discussion

Compared with the pathological examination, detecting markers in whole blood after surgery has many advantages (25). The previous studies demonstrated that many clinical test indicators have diagnostic and prognostic functions in the diagnosis and metastasis of BC. A meta-analysis with 12,993 subjects showed that elevated serum CA153 or CEA was associated with poor overall and disease-free survival in BC patients (26). Serums CEA, CA199, CA125, CA153, and TPS can diagnose metastatic BC, and different combinations of tumor markers have varying diagnostic values (22).

The present study examined a cohort of 938 BC patients and 509 benign breast disease patients which investigated the association between peripheral blood NLR, LMR, PLR, and other traditional markers. Moreover, the role of these indicators on diagnosis and treatment effects in BC was explored. Our observations on NLR, LMR, and PLR are consistent with the research on multiple cancer types, including BC, in recent years (15, 16, 27–34).

Next, we analyzed the diagnostic accuracy of all indicators. Many previous studies have shown that the threshold is based on the prognosis or quartile of the analyzed cohort (35, 36). Our study used a pooled database of matched patients with stage I-III BC patients and stage IV BC patients. This strategy highlighted significantly higher CEA, CA153, CA125, and killer T cell values in stage I-III BC patients compared to stage IV BC patients. LMR had lower values in stage IV BC patients. It enabled us to compute a threshold based on diseases’ progress and be different from the results obtained by benign disease patients as the control cohort. The obtained cut-offs for CEA, CA153, CA125, killer T cell, and LMR are slightly higher for those found in previous studies (CEA: 5 ng/ml, CA153: 31.3 U/ml, B cell%: literature range: 7%–23%, Th/Tc: literature range: 0.9-3.6, LMR: literature range: 3-5.5) (20, 30–32, 37–39). ROC analysis showed that the accuracy of a single marker for diagnosis of stage I-III BC patients and stage IV BC patients was not high (AUC <0.800). The combined use of CEA, CA153, CA125, killer T cell, and LMR can greatly enhance the diagnostic ability compared to using a single indicator, which indicates that this combination can better predict metastasis risk of BC (AUC =0.829).

Although many previous studies have reported the predictive utility of NLR, LMR, and PLR in treating BC patients, the results obtained are not consistent. Cho U’s (40) reviewed 661 patients diagnosed with invasive BC from 1993 to 2011. In univariate analysis, high NLR, PLR, and low LMR are significantly associated with poor DSS and DFS. In multivariate analysis, only PLR is still considered as an independent predictor of prognosis. In gastrointestinal cancer, BC, and gynecological cancer, multivariate Cox regression analysis found that high expression of NLR was independently associated with decreased PFS (41). In a study of the effect of circulating blood lymphocyte subsets on the survival of patients with metastatic BC (MBC), Th cells was a negative independent predictor of PFS (hazard ratio [HR] = 0.538, 95% confidence interval [CI] = 0.313-0.926, P = 0.025) (42). Both Th and Tc increase and participate in the immune response. Tc cells are the key effector cell population that mediates effective anti-tumor immunity (43, 44). On the contrary, Th cells in the tumor have a negative prognostic effect on the prognosis of BC patients.

The degree of tumor malignancy is related to non-specific inflammation. Inflammatory mediators can cause abnormal proliferation and deterioration of tumor cells (45). This study used metastatic BC with a high degree of malignancy to divide the cut-off value of LMR and other hematological indicators. It used this value to find effective markers to predict the risk of distant metastasis and stage I- III BC prognosis. Although different cut-off values were selected, consistent with our research is that LMR predicts the efficacy and prognosis of BC patients (16, 20, 46). However, relevant research reports also have different results. The clinical prognostic effect of PLR in BC is better than that of LMR (47).

We followed up a larger BC cohort and included tumor proliferation, cellular immunity, and inflammatory factors before treatment compared with other studies. We were surprised to find that the Th/Tc (CD4:CD8) ratio is an independent prognostic factor for stage I- III BC. Th/Tc ratio repeatedly reports being associated with lymph node metastasis and the prognosis of triple-negative BC (44, 48–52).

However, this study has limitations. First of all, the patients enrolled in this study are single-center, which lacks the universality of the results. Second, due to the limited follow-up data, detailed treatment factors did not include in the analysis (including neoadjuvant chemotherapy, immunotherapy, and surgical methods), which may affect the accuracy of the results. Third, because there is no epidemiological investigation of patients, the level of peripheral blood markers may be affected by surgical methods and accompanying diseases. Finally, this study may help better understand the relationship between different types of whole blood markers and BC progress.

## Conclusions

The prognosis of BC recurrence and metastasis is poor, and there is an urgent need for easily available predictors. This study found that low LMR and Th/Tc ratios in stage I- III BC indicate poor prognosis. Additionally, LMR combined with other indicators (CEA, CA153, CA125, and Tc cell%) can enhance the predictive value of BC distant metastasis. Although CEA, CA153, CA125, NLR, PLR, and other factors are not independent prognostic indicators of DFS, the values of NLR and PLR are related to TNM staging. CEA, CA153, and CA125 can independently predict metastasis, suggesting that other markers still have clinical significance. We hope this research can help doctors treat BC patients.

## Data Availability Statement

The original contributions presented in the study are included in the article/**Supplementary Material**. Further inquiries can be directed to the corresponding author.

## Ethics Statement

The studies involving human participants were reviewed and approved by Guangxi Medical University Cancer Hospital Ethics Committee. The patients/participants provided their written informed consent to participate in this study.

## Author Contributions

HL and LZ designed the study. ZZ, YC, WL, DW and CS performed the experiments. HL and ZZ analyzed the data. ZZ and QL wrote the manuscript. All authors approved the manuscript.

## Funding

This study support by grants from the Scientific Research & Technical Development Project of Qingxiu District, Nanning City, Guangxi Province (No. 2017036; No. 2016051). The Guangxi Scientific Research and Technical Planning Project (Grant No. Guike AB19110018). Self-funded scientific research project of Guangxi Zhuang Autonomous Region Health Committee (Grant No. 20191022).

## Conflict of Interest

The authors declare that the research was conducted in the absence of any commercial or financial relationships that could be construed as a potential conflict of interest.

## Publisher’s Note

All claims expressed in this article are solely those of the authors and do not necessarily represent those of their affiliated organizations, or those of the publisher, the editors and the reviewers. Any product that may be evaluated in this article, or claim that may be made by its manufacturer, is not guaranteed or endorsed by the publisher.

## Abbreviations

BC, breast cancer; EGFR, epidermal growth factor receptor; CEA, carcinoembryonic antigen; CA125, carbohydrate antigen 125; CA153, carbohydrate antigen 153; NK, natural killer; NEUT, neutrophile; LYMPH, lymphocyte; MONO, monocyte; PLT, platelet; NLR, neutrophil to lymphocyte ratio; LMR, lymphocyte to monocyte ratio; Th, helper T cell; Tc, killer T cell; Th/Tc ratio, helper T cell to killer T cell ratio; CTC, circulating tumor cells; ROC, receiver operating characteristic; LASSO, the minor absolute shrinkage, and selection operator; PFS, progression-free survival; CI, confidence interval; OR, odds ratio; AUC, the area under curve; HR, hazard ratio.
