# Supplementary material for: Prognostic Evaluation of Metastasis-Related Lymphocyte/Monocyte Ratio in Stage Ⅰ-Ⅲ Breast Cancer Receiving Chemotherapy
Source: Front Oncol. 2022 Mar 24;11:782383. doi: 10.3389/fonc.2021.782383 (PMC8987500; doi:10.3389/fonc.2021.782383)
Supplement: Supplementary file 1 [file DataSheet_1.pdf]

Prognostic evaluation of metastasis-related lymphocyte/monocyte ratio in stage I-III breast cancer receiving chemotherapy

Supplementary

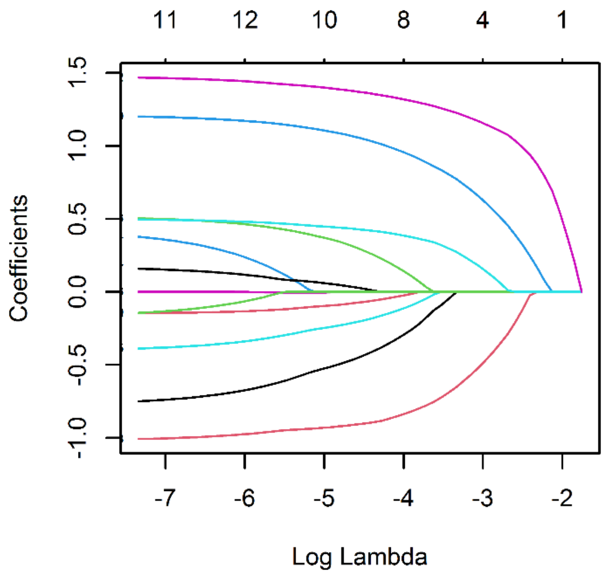

Figure S1. LASSO model shows that the significant four factors for BC diagnosis include Tcell, helper T cell, killer T cell, B cell, NLR, LMR, CEA, CA125, and CA153.

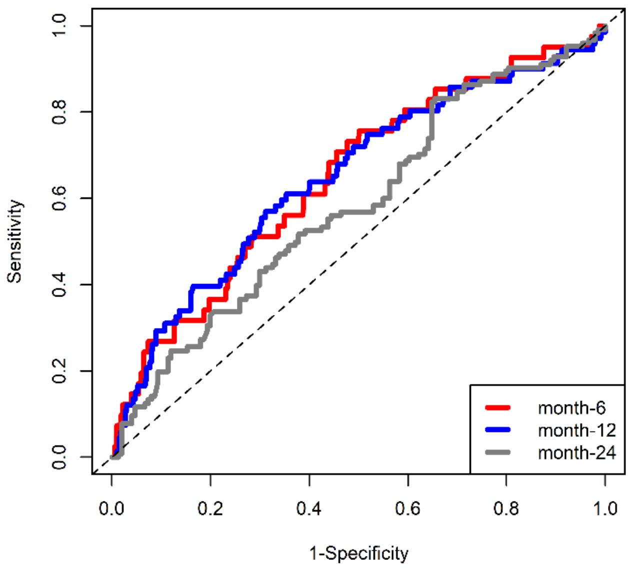

Figure S2. Time-dependent ROC curves of the LMR in BC. The red line represents the 6-month prognosis, the blue line represents the 12-month prognosis, and the gray line represents the 24-month prognosis.

Table S1

The correlation of peripheral blood index with onset and metastasis analyzed by ROC in BC

	Onset					Metastasis				
	**AUC (95%CI)**	**threshold**	**specificity**	**sensitivity**	**Youden index**	**AUC (95%CI)**	**threshold**	**specificity**	**sensitivity**	**Youden index**
CA153						**0.793 (0.755-0.832)**	21.85	0.827	0.649	**0.477**
CEA						***0.751 (0.709-0.792)***	*2.485*	*0.742*	**0.66**	*0.401*
CA125						**0.696 (0.651-0.741)**	22.75	0.771	0.534	0.305
LMR	0.574 (0.543-0.606)	**5.239**	0.554	0.576	0.13	***0.663 (0.616-0.709)***	*3.433*	***0.846***	*0.424*	*0.27*
NLR	0.519 (0.487-0.551)	**1.711**	0.678	0.39	0.067	**0.634 (0.588-0.679)**	2.543	0.728	0.487	0.215
PLR	0.507 (0.475-0.539)	**237.011**	0.949	0.112	0.061	*0.581 (0.534-0.629)*	*195.307*	*0.786*	*0.356*	*0.142*
Th cell%						0.573 (0.526-0.621)	32.575	0.831	0.314	0.145
Th/Tc radio						*0.553 (0.506-0.601)*	*1.946*	*0.505*	*0.602*	*0.107*
T cell%						0.546 (0.498-0.594)	53.315	0.933	0.183	0.116
Tc cell%						*0.524 (0.475-0.572)*	*21.235*	*0.545*	*0.545*	*0.089*
B cell%						0.523 (0.474-0.572)	8.715	0.794	0.319	0.113
NK cell%						*0.491 (0.445-0.538)*	*3.6*	*0.967*	*0.079*	*0.045*

Abbreviation: CEA, carcinoembryonic antigen; CA125, carbohydrate antigen 125; CA153, carbohydrate antigen 153; NK, natural killer; LYMPH, lymphocyte; PLT, platelet; NLR, neutrophil to lymphocyte ratio; PLR, platelet to lymphocyte ratio; LMR, lymphocyte to monocyte ratio; Th/Tc ratio, helper T cell to killer T cell ratio; AUC, area under the curve.

Table S2
Associations of clinicopathological features with inflammatory biomarkers in breast cancer. (n =938).

Variables	*LMR*			*NLR*			*PLR*		
	<3.433	>=3.433	p	<2.543	>=2.543	p	<195.307	>=195.307	p
Age (years)			0.419			0.326			0.936
<45	72	237		205	104		232	77	
⩾45 to <55	70	276		247	99		264	82	
⩾55	54	229		190	93		214	69	
T			**<0.001 ***			**<0.001 ***			**0.001**
T1-T2	113	558		483	188		528	143	
T3-T4	83	184		159	108		182	85	
N			**0.018 ***			**0.020 ***			**0.036**
N0	71	339		297	113		324	86	
N1-NX	125	403		345	183		386	142	
M			**<0.001 ***			**<0.001 ***			**<0.001 ***
M0	115	632		544	203		587	160	
M1	81	110		98	93		123	68	
TNM stage			**<0.001 ***			**0.005 ***			**0.001**
Ⅰ - Ⅱ	82	472		399	155		441	113	
Ⅲ- Ⅳ	114	270		243	141		269	115	
Molecular classification			0.616			0.888			0.598
luminalA	20	75		68	27		75	20	
luminalB(HER2(-))	51	231		196	86		218	64	
luminalB(HER2(+))	72	268		229	111		251	89	
ERBB2	29	90		82	37		86	33	
Triple	24	78		67	35		80	22	
Progesterone Receptor			0.329			0.926			0.955
-	69	234		208	95		229	74	
+	127	508		434	201		481	154	
Estrogen Receptor			0.119			0.523			0.786
-	65	204		180	89		202	67	
+	131	538		462	207		508	161	
HER2			0.414			0.657			0.112
-	95	384		331	148		373	106	
+	101	358		311	148		337	122	

Ki-67			0.584			0.512			0.982
<14%	36	124		106	54		121	39	
≧14%	160	618		536	242		589	189	
CK5/6			0.946			0.864			0.584
-	171	646		560	257		616	201	
+	25	96		82	39		94	27	
EGFR			0.284			0.247			0.140
-	156	615		534	237		591	180	
+	40	127		108	59		119	48	

Note: χ2 test

Abbreviation: EGRF epidermal growth factor receptor, CEA carcinoembryonic antigen, CA125 carbohydrate antigen 125, CA153 carbohydrate antigen 153, Th helper T cell, Tc killer T cell, NK natural killer, NEUT neutrophile, LYMPH lymphocyte, MONO monocyte, PLT platelet, NLR neutrophil-lymphocyte ratio, LMR lymphocyte-monocyte ratio, PLR platelet-lymphocyte ratio.

Table S3

DFS of the Stage IV BC patients based on univariate and multivariate Cox proportional regression analyses.

Profiles	DFS						
	Univariate Cox regression				Multivariare Cox regression		
	HR	95(%)CI		p value	HR	95(%)CI	p value
Bcell>=8.715	0.7216	0.4244	1.2270	0.2280			
CA125>=22.75	1.2320	0.7306	2.0790	0.4330			
CA153>=21.850	0.9222	0.5362	1.5860	0.7700			
CEA>=2.485	1.5450	0.8679	2.7490	0.1390			
CK5/6+	0.8657	0.3924	1.9100	0.7210			
EGFR+	0.4958	0.1981	1.2410	0.1340			
ER+	0.7563	0.4484	1.2760	0.2950			
HER2+	1.0270	0.6133	1.7200	0.9190			
Ki-67+	0.8528	0.4592	1.5840	0.6140			
LMR>=3.433	0.8903	0.5301	1.4950	0.6600			
N1-NX	1.6690	0.7570	3.6800	0.2040			
NK>=3.6	0.5098	0.2306	1.1270	0.0959			
NLR>=2.543	1.0290	0.6146	1.7240	0.9120			
PLR>=195.307	0.7972	0.4631	1.3720	0.4130			
PR+	0.7615	0.4546	1.2760	0.3010			
T3-T4	0.9937	0.5889	1.6770	0.9810			
Tc>=21.235	0.7603	0.4527	1.2770	0.3000			
Tcell>=53.315	0.7606	0.4102	1.4100	0.3850			
Th_Tc_ratio>=1.946	1.4460	0.8582	2.4360	0.1660			
Th>=32.575	1.0340	0.5975	1.7910	0.9040			

Abbreviation: CEA, carcinoembryonic antigen; CA125, carbohydrate antigen 125; CA153, carbohydrate antigen 153; NK, natural killer; NLR, neutrophil to lymphocyte ratio; PLR, platelet to lymphocyte ratio; LMR, lymphocyte to monocyte ratio; Th/Tc ratio, helper T cell to killer T cell ratio; CI, confidence interval; HR, hazard ratio.

Table S4

Univariate and multivariate cox regression analysis on different molecular subtypes of BC cancer

molecular subtypes	Univariate					Multivariate			
	**Profiles**	**OR**	**95 (%) CI**		**p**	**OR**	**95 (%) CI**		**p**
HR-positive (N=585)	Bcell>=8.715	0.4402	0.1814	1.068	0.07				
	CA125>=22.75	2.053	0.8375	5.033	0.116				
	CA153>=21.850	0.971	0.3242	2.908	0.958				
	CEA>=2.485	1.162	0.4459	3.029	0.758				
	CK5-6+	1.381	0.3194	5.969	0.666				
	EGFR+	2.175	0.7898	5.988	0.133				
	HER2+	1.667	0.6804	4.085	0.264				
	Ki 67+	2.714	0.6272	11.74	0.182				
	**LMR>=3.433**	**0.395**	**0.157**	**0.992**	**0.048**	**0.427**	**0.17**	**1.077**	**0.071**
	N1-NX	1.539	0.6126	3.864	0.359				
	NK>=3.6	0.5841	0.07814	4.367	0.6				
	NLR>=2.543	2.327	0.9621	5.627	0.061				
	PLR>=195.307	1.242	0.4763	3.238	0.658				
	T3-T4	1.417	0.5435	3.693	0.476				
	Tc>=21.235	1.622	0.6624	3.973	0.29				
	Tcell>=53.315	0.431	0.1262	1.472	0.179				
	Th/Tc ratio>=1.946	0.4552	0.1747	1.186	0.107				
	**Th>=32.575**	**0.3382**	**0.1348**	**0.8481**	**0.021**	**0.362**	**0.144**	**0.911**	**0.031**
HR-negative (N=162)	Bcell>=8.715	2.442	0.306	19.48	0.399				
	CA125>=22.75	1.435	0.403	5.108	0.578				
	CA153>=21.850	1.935	0.498	7.513	0.34				
	CEA>=2.485	0.73	0.155	3.444	0.691				
	CK5-6+	0.302	0.038	2.395	0.257				
	EGFR+	1.54	0.446	5.322	0.495				
	HER2+	0.858	0.248	2.968	0.809				
	Ki 67+	74411087	0	inf	0.999				
	**LMR>=3.433**	**0.288**	**0.081**	**1.022**	**0.054**				
	**N1-NX**	**4.596**	**0.975**	**21.67**	**0.054**				
	NK>=3.6	25533978	0	inf	0.998				
	NLR>=2.543	0.693	0.147	3.272	0.643				
	PLR>=195.307	0.877	0.186	4.14	0.869				
	T3-T4	1.449	0.37	5.67	0.594				
	Tc>=21.235	2.77	0.713	10.76	0.141				
	Tcell>=53.315	25985789	0	inf	0.998				
	**Th/Tc ratio>=1.946**	**0.113**	**0.014**	**0.89**	**0.038**				
	Th>=32.575	0.302	0.085	1.075	0.065				
Her2-positive	Bcell>=8.715	1.197	0.387	3.702	0.755				

(N=360)	CA125>=22.75	1.133	0.399	3.221	0.815				
	CA153>=21.850	1.471	0.478	4.528	0.501				
	CEA>=2.485	1.041	0.367	2.958	0.939				
	CK5/6+	0	0	#VALUE!	0.998				
	EGFR+	2.382	0.88	6.451	0.088				
	ER+	0.592	0.225	1.558	0.289				
	Ki 67+	1.151	0.26	5.104	0.853				
	LMR>=3.433	0.509	0.179	1.447	0.205				
	**N1-NX**	**4.483**	**1.287**	**15.62**	**0.019**	**4.269**	**1.225**	**14.884**	**0.023**
	NK>=3.6	0.512	0.068	3.866	0.516				
	NLR>=2.543	1.608	0.591	4.374	0.352				
	PLR>=195.307	0.925	0.301	2.842	0.892				
	PR+	0.468	0.18	1.214	0.119				
	T3-T4	2.11	0.802	5.552	0.13				
	Tc>=21.235	2.391	0.84	6.807	0.102				
	Tcell>=53.315	0.328	0.074	1.45	0.142				
	Th_Tc_ratio>=1.946	0.349	0.113	1.073	0.066				
	**Th>=32.575**	**0.316**	**0.117**	**0.854**	**0.023**	**0.3402**	**0.1256**	**0.9218**	**0.034**
Her2-negative (N=387)	Bcell>=8.715	0.361	0.12	1.081	0.069				
	**CA125>=22.75**	**3.544**	**1.186**	**10.59**	**0.024**	**2.873**	**0.939**	**8.79**	**0.064**
	CA153>=21.850	1.135	0.309	4.169	0.849				
	CEA>=2.485	0.951	0.261	3.458	0.939				
	CK5/6+	1.707	0.467	6.238	0.419				
	EGFR+	1.956	0.602	6.36	0.265				
	ER+	0.438	0.143	1.342	0.148				
	Ki 67+	286300787	0	inf	0.998				
	**LMR>=3.433**	**0.223**	**0.075**	**0.666**	**0.007**	**0.271**	**0.089**	**0.827**	**0.022**
	N1-NX	1.054	0.351	3.168	0.925				
	NK>=3.6	25545146	0	inf	0.998				
	NLR>=2.543	1.706	0.558	5.219	0.349				
	PLR>=195.307	1.415	0.433	4.622	0.565				
	PR+	0.476	0.155	1.467	0.196				
	T3-T4	0.681	0.151	3.082	0.618				
	Tc>=21.235	1.441	0.484	4.295	0.512				
	Tcell>=53.315	1.101	0.143	8.488	0.926				
	Th_Tc_ratio>=1.946	0.288	0.079	1.048	0.059				
	Th>=32.575	0.339	0.111	1.038	0.058				
triple-negative (N=77)	Bcell>=8.715	1.068	0.119	9.592	0.953				
	CA125>=22.75	1.299	0.216	7.827	0.775				
	CA153>=21.850	0	0	inf	0.999				
	CEA>=2.485	0.829	0.092	7.431	0.867				
	CK5-6+	0.267	0.03	2.392	0.238				

EGFR+	0.694	0.116	4.168	0.69
Ki 67+	84856963	0	inf	0.999
LMR>=3.433	0.293	0.049	1.773	0.182
N1-NX	1.81	0.302	10.84	0.516
NK>=3.6	25652372	0	inf	0.999
NLR>=2.543	0.546	0.061	4.89	0.589
PLR>=195.307	0	0	inf	0.999
T3-T4	1.038	0.115	9.357	0.973
Tc>=21.235	1.39	0.232	8.336	0.719
Tcell>=53.315	26185915	0	inf	0.999
Th/Tc ratio>=1.946	0.302	0.034	2.717	0.286
Th>=32.575	0.305	0.05	1.861	0.198

Abbreviation: CEA, carcinoembryonic antigen; CA125, carbohydrate antigen 125; CA153, carbohydrate antigen 153; NK, natural killer; NLR, neutrophil to lymphocyte ratio; PLR, platelet to lymphocyte ratio; LMR, lymphocyte to monocyte ratio; Th/Tc ratio, helper T cell to killer T cell ratio; CI, confidence interval; HR, hazard ratio.

Supplementary Methods

The experimental methods of cellular immunology related indicators

Flow cytometry assay was used to detect T cells, B cells, and NK cells. First, the cells are stained according to the operating instructions, and antibodies against CD8, CD19, CD3, CD4, and NK cells are directly added to the bottom of each test tube. In the second step, 100 μL of the mixed whole blood is directly added to the bottom of the test tube, after gently shaking, the test tube is placed in a dark condition at 4° C for 30 minutes, to allow various antibodies to fully bind to the corresponding receptor. Then, the red blood cell lysis buffer is added, shaken gently to lyse the blood cells in the sample, and the sample is placed in the dark. After 10 minutes, add 1.5 mL PBS to stop the lysis of red blood cells, and place the test tube in a shaker with gentle shaking. After the sample was separated by an automatic separation device at 350 g and centrifuged for 5 minutes, the flow tube was removed, the supernatant was discarded, and only the precipitate was retained. 0.5 ml cell suspension was added to surface label cell fixing, the samples were stored at room temperature for 20 minutes, after that, another 350 g 5 minutes centrifugation was needed. Then discard the supernatants and add 2 ml film-breaking agent, centrifugate at 350 g for 10-min. Mix the intracellular labeled monoclonal antibody in the sample and place it at 4° C for 30 minutes, then wash twice with an intracellular labeled monoclonal antibody and centrifuge, discard the supernatant, and 0.5ml PBS was added for cell suspension. Finally, flow cytometry (BD Biosciences, Franklin Lakes, NJ, USA) was done to detect cells and analyze the results.
